# Histamine H_2_-Receptor Antagonists Improve Non-Steroidal Anti-Inflammatory Drug-Induced Intestinal Dysbiosis

**DOI:** 10.3390/ijms21218166

**Published:** 2020-10-31

**Authors:** Rei Kawashima, Shun Tamaki, Fumitaka Kawakami, Tatsunori Maekawa, Takafumi Ichikawa

**Affiliations:** Department of Regulation Biochemistry, Kitasato University Graduate School of Medical Sciences, Kanagawa 252-0374, Japan; rkawa@kitasato-u.ac.jp (R.K.); mm19024@st.kitasato-u.ac.jp (S.T.); kawakami@kitasato-u.ac.jp (F.K.); maekawa@kitasato-u.ac.jp (T.M.)

**Keywords:** H_2_RA (histamine H_2_ receptor antagonist), NSAIDs (non-steroidal anti-inflammatory drugs), dysbiosis, intestinal flora, organic acid, feces

## Abstract

Dysbiosis, an imbalance of intestinal flora, can cause serious conditions such as obesity, cancer, and psychoneurological disorders. One cause of dysbiosis is inflammation. Ulcerative enteritis is a side effect of non-steroidal anti-inflammatory drugs (NSAIDs). To counteract this side effect, we proposed the concurrent use of histamine H_2_ receptor antagonists (H_2_RA), and we examined the effect on the intestinal flora. We generated a murine model of NSAID-induced intestinal mucosal injury, and we administered oral H_2_RA to the mice. We collected stool samples, compared the composition of intestinal flora using terminal restriction fragment length polymorphism, and performed organic acid analysis using high-performance liquid chromatography. The intestinal flora analysis revealed that NSAID [indomethacin (IDM)] administration increased *Erysipelotrichacea*e and decreased *Clostridiales* but that both had improved with the concurrent administration of H_2_RA. Fecal levels of acetic, propionic, and n-butyric acids increased with IDM administration and decreased with the concurrent administration of H_2_RA. Although in NSAID-induced gastroenteritis the proportion of intestinal microorganisms changes, leading to the deterioration of the intestinal environment, concurrent administration of H_2_RA can normalize the intestinal flora.

## 1. Introduction

Dysbiosis (imbalance) refers to the state of disturbance in the balance of the microbial flora in the body, which allows illness to develop in the host and can cause collapse of the environment in the body established through coexistence to date [1]. In particular, gastroenteritis is often caused by dysbiosis. Reported cases have revealed the onset of obesity, autoimmune disease, cancer, and psychoneurological disorders [2,3,4]. Recently, many publications have indicated a relationship between inflammation and bacterial flora because dysbiosis induces failure of immunological homeostasis and causes inflammatory bowel disease (IBD) [5,6], and the intestinal flora controls the secretion of inflammatory cytokines and inhibits the onset of diabetes [7].

Non-steroidal anti-inflammatory drugs (NSAIDs) inhibit the synthesis of prostaglandin E_2_, which is a phlogogenic and pain-intensifying substance, by inhibiting cyclooxygenase (COX) of the arachidonic acid cascade, thereby exhibiting analgesic, fever-lowering, and anti-inflammatory actions [8]. Unfortunately, conventional NSAIDs (nonselective NSAIDs) inhibit not only COX-2 but also COX-1, which is necessary for maintaining organ homeostasis, thereby causing side effects such as gastrointestinal disturbance and renal impairment [9]. As a countermeasure, the concurrent use of proton pump inhibitors (PPIs), which are considered gastroprotective agents, has been adopted to alleviate NSAID-induced enteritis accompanied with peptic ulcers and bleeding [10,11,12]. However, reports have demonstrated that concurrent treatment using NSAIDs and PPIs, exacerbates enteritis [13] and inhibits intestinal motility and that it was unable to prevent bacteria proliferation due to inflammation [14]. Histamine H_2_-receptor antagonists (H_2_RA) have a competitive antagonistic action at histamine H2 receptors located in parietal cells of the stomach, and like PPIs, they inhibit gastric acid secretion. While PPIs have a more potent inhibitory action than H_2_RA, they change the composition of the bacterial flora and potentially increase the risk of enteral infection [15,16]; therefore, it is perceived that H_2_RA can be used instead of PPI as an anti-inflammatory agent that is compatible with NSAIDs. Previously, our group reported that lafutidine, a second-generation H_2_RA, activated mucus-secreting cells [17,18] and could effectively prevent mucositis caused by cancer chemotherapy [19]. Based on these reports, we inferred that H_2_RA contributes to the barrier against exogenous microorganisms. Therefore, to evaluate the effect of H_2_RA against dysbiosis caused by enteritis, we analyzed the microbiome of a murine model of NSAID-induced mucosal injury.

## 2. Results

### 2.1. Biological Changes

**Figure 1:**
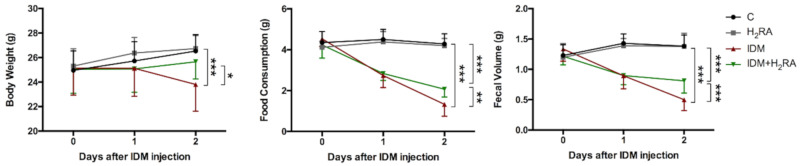
Biological changes in Indometacin (IDM)-induced intestinal mucosal injury model with or without histamine H_2_-receptor antagonists (H_2_RA) administration. Fifty mice (16 control, 5 H_2_RA only, 13 IDM, and 16 IDM + H_2_RA mice) were administered a single dose of IDM and were administered H_2_RA daily. From the first day of IDM administration, defined as day 0, body weight, food intake, and fecal volume were measured daily. Statistical analysis was performed using two-way analysis of variance (ANOVA) with Tukey’s post-hoc test. * *p* < 0.05, ** *p* < 0.01, *** *p* < 0.001.

To determine the effects of Indometacin (IDM) and histamine H_2_-receptor antagonists (H_2_RA) on the whole body, we measured the bodyweight of each group over time. In the control group (C), bodyweight tended to increase daily. In the IDM group, body weight decreased by approximately 5.2% on day 2 post administration compared with pre-administration. In the IDM concurrent with the H_2_RA (IDM + H_2_RA) group, this decrease in body weight was inhibited (Figure 1). Furthermore, to evaluate gastrointestinal absorption, a comparison of food intake and fecal volume between each group was made. There was no change in food intake in group C between the pre-and post-administration states; however, two days after IDM administration, food intake decreased by 71% in the IDM group. The decrease in food intake in the IDM + H_2_RA group was maintained at 50% (Figure 1). The fecal volume for group C showed almost no change between the pre-and post-administration states; however, two days after IDM administration fecal volume in the IDM group decreased by 63% and by 33% in the IDM + H_2_RA group (Figure 1).

Therefore, we found that the administration of IDM affected the body with a decrease in body weight, food intake, and fecal volume; however, these results improved with the concurrent use of H_2_RA, suggesting that H_2_RA helped restore basic bodily functions.

### 2.2. Fecal Properties

**Figure 2:**
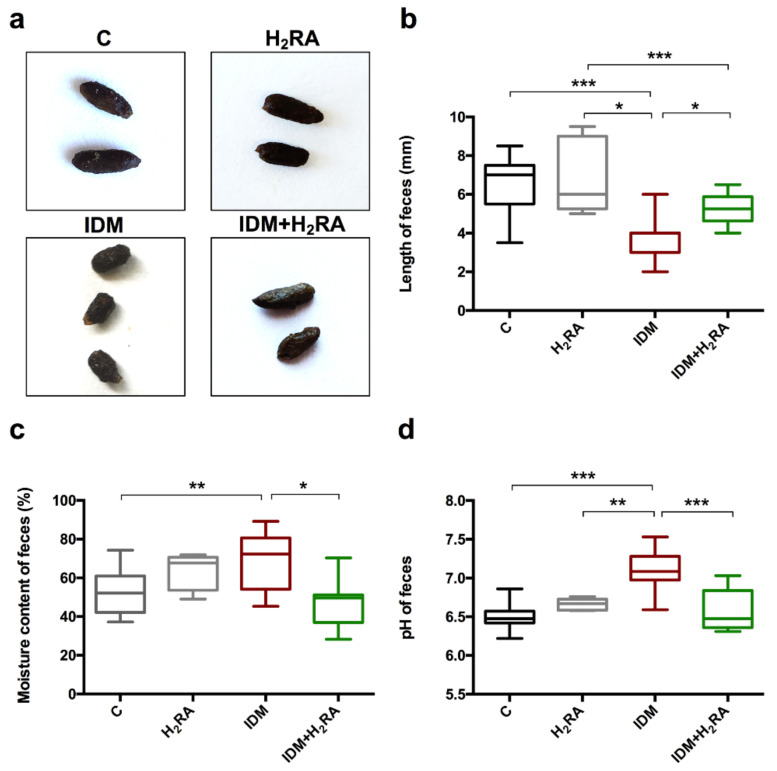
State of feces in Indometacin (IDM)-induced intestinal mucosal injury model with or without histamine H_2_-receptor antagonists (H_2_RA) administration. Fecal samples were collected from the 50 mice (16 control, 5 H_2_RA only, 13 IDM, and 16 IDM + H_2_RA mice) on day 2 after IDM administration. We examined macroscopic observations (**a**), length per feces (**b**), moisture content (**c**), and pH (**d**). Statistical analysis was performed using two-way ANOVA with Tukey’s post-hoc test. * *p* < 0.05, ** *p* < 0.01, *** *p* < 0.001.

To examine the effects of IDM and H_2_RA on feces subject to this experiment, we observed fecal properties. Macroscopic observation revealed no particular differences in fecal color and shine between each group (Figure 2a). However, the fecal form was relatively short in the IDM group, and the same length was noted in the C group, H_2_RA group, and IDM + H_2_RA group (Figure 2a,b). The moisture content of the feces was elevated in the IDM group but low in the IDM + H_2_RA group (Figure 2c). In the group administered H_2_RA alone, the moisture content somewhat increased; however, no significant difference was observed. The fecal pH level was slightly acidic at close to 6.5 in the C group, shifted to approximately 7.1 in the IDM group, and returned to the slightly acidic side comparable with group C in the IDM + H_2_RA group (Figure 2d). By determining the fecal properties, it is conceivable that the concurrent use of H_2_RA is related to the intestinal environment.

### 2.3. Composition of the Intestinal Flora

**Figure 3:**
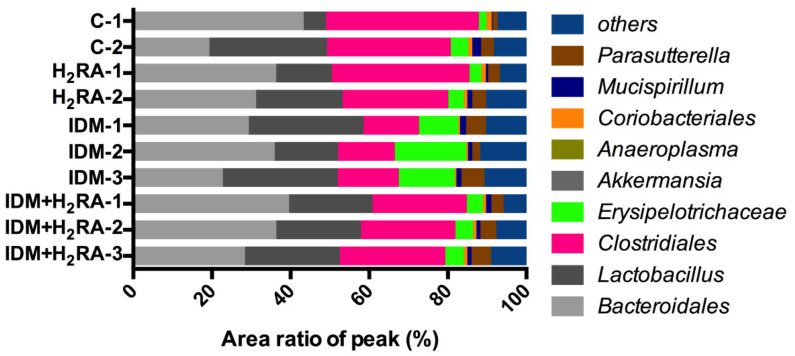
Constitution of intestinal flora in the feces of Indometacin (IDM)-induced intestinal mucosal injury model with or without histamine H_2_-receptor antagonists (H_2_RA) administration. On the second day after IDM administration, we conducted a T-RFLP using the feces of 10 mice (2 control, 2 H_2_RA only, 3 IDM, and 3 IDM + H_2_RA mice). The area ratio of peaks of the bacterial classification groups corresponding to each operational taxonomic unit (OTU) was presented for each classification group. The final number indicates the individual mouse number.

To examine the effects of IDM and H_2_RA on the intestinal flora, we conducted a T-RFLP on the fecal samples obtained two days after IDM administration. The peak detected from each specimen was treated as operational taxonomic unit (OTU), and we calculated the area ratio of T-RFLP peaks of each OTU. The area ratio of peaks for the bacterial taxa corresponding to each OTU was summarized for each classification group and was presented in a cumulative bar chart (Figure 3). The group classification was inferred based on the murine intestinal flora database created by the Central Institute for Experimental Animals (CIEA). Furthermore, unknown OTU and presumed bacterial groups were considered unclassified bacteria defined as others. The number of samples was analyzed in two animals of group C and group H_2_RA and in three animals of the IDM group and IDM + H_2_RA group. When performing statistical tests to determine a significant difference, the number *n* was slightly smaller; however, in the intestinal flora analysis, on consolidating the data of each mouse, it is inappropriate to speak of a significant difference. Therefore, we conducted an experiment comprising several animals selected among mice in which a model was rigorously created.

Figure 3 shows that for the estimated area ratio of peaks for the *Erysipelotrichaceae* family (green), the mean value was 3.19% in group C, 3.49% in the H_2_RA group, 14.16% in the IDM group, and 4.46% in the IDM + H_2_RA group. Therefore, the *Erysipelotrichaceae* family in feces increased with IDM administration, and decreased with concurrent H_2_RA. That is, the H_2_RA group showed comparable values to group C, and when H_2_RA was administered alone, there was nearly no impact on the bacterial flora composition.

Furthermore, for the estimated area ratio of peaks for bacteria of the *Clostridiales* order (pink), the mean value was 35.16% for the C group, 30.94% for the H_2_RA group, 14.69% for the IDM group, and 24.94% for the IDM + H_2_RA group. Therefore, the amount of *Clostridiales* order in feces increased with IDM administration and decreased with concurrent H_2_RA. That is, the H_2_RA group showed comparable values to group C, and when H_2_RA was administered alone, there was nearly no impact on the bacterial flora composition. For the species other than the two types of bacterial flora noted above, there was no major change observed between the four groups.

### 2.4. The Inter-Specimen Difference in Bacterial Flora

**Figure 4:**
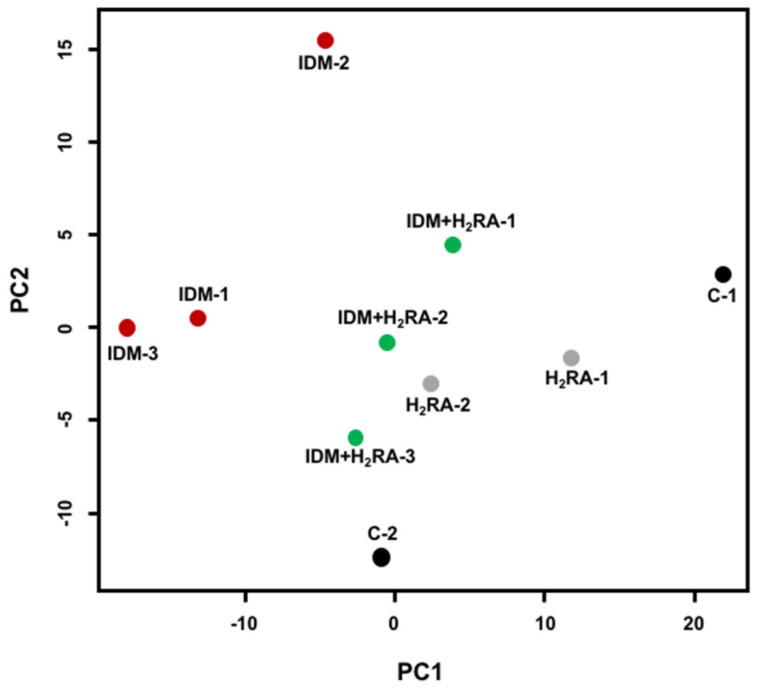
Conversion to 2D chart of intestinal flora classification in the feces of Indometacin (IDM)-induced intestinal mucosal injury model with or without histamine H_2_-receptor antagonists (H_2_RA) administration. Based on the data of the area ratio of peaks for bacterial taxa (Figure 3), analysis was performed using principal coordinates analysis (PCoA). The final number indicates the individual mouse number.

Based on the data obtained regarding the area ratio of peaks, we conducted a two-dimensional visualization analysis using principal coordinates analysis (PCoA). First, between samples within each group, relatively similar coordinates were plotted. Therefore, we believe that individual differences within each group were adequately suppressed, and that the data were reliable in terms of intestinal flora constitution (Figure 4).

On comparing the coordinates between groups, the regions of IDM group plots were extremely distant to those of groups C and H_2_RA, whereas the plots of the IDM + H_2_RA group approached the regions of groups C and H_2_RA (Figure 4). Therefore, it was suggested that IDM administration causes a deviation in the bacterial flora, and with concurrent use of lafutidine, the bacterial flora is normalized.

### 2.5. The Effect on Organic Acid Content

**Figure 5:**
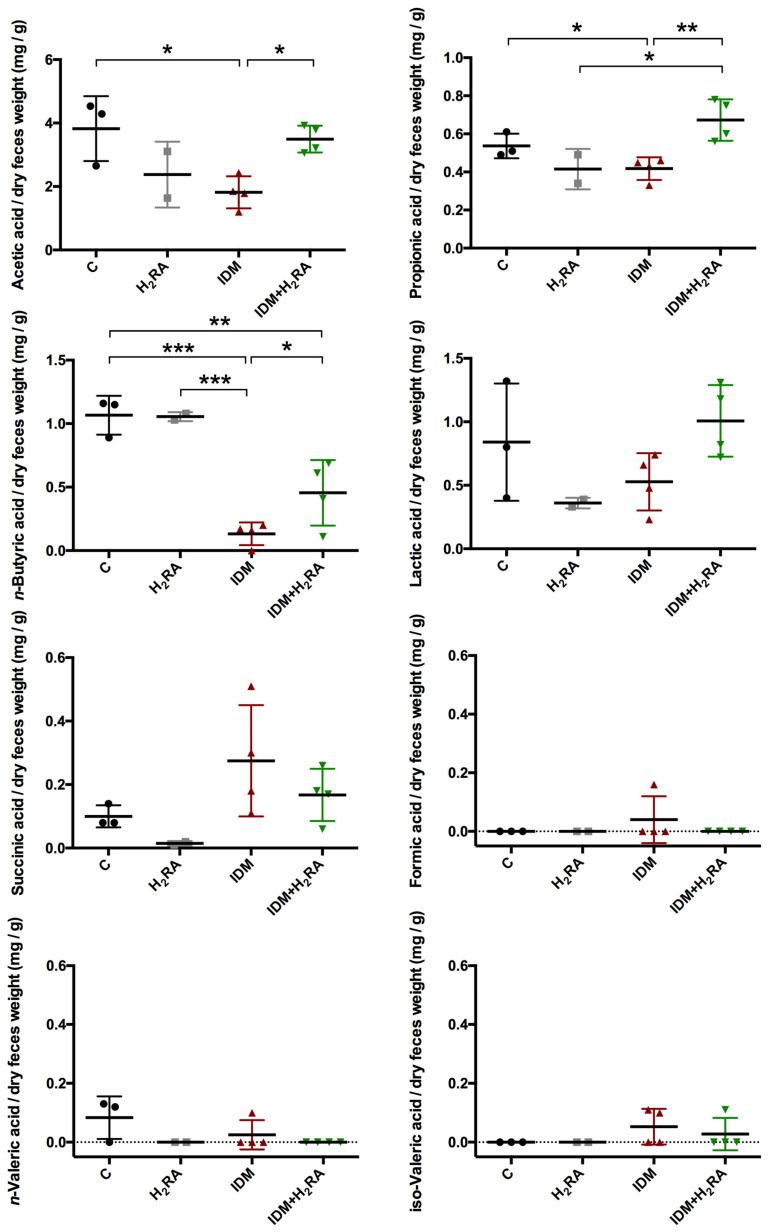
Effect on organic acid contents in the feces of Indometacin (IDM)-induced intestinal mucosal injury model with or without histamine H_2_-receptor antagonists (H_2_RA) administration. Two days after IDM administration, organic acids (acetic acid, propionic acid, *n*-butyric acid, lactic acid, succinic acid, formic acid, *n*-valeric acid, and iso-valeric acid) were measured using the feces of 10 mice (3 control, 2 H_2_RA only, 4 IDM, and 4 IDM + H_2_RA mice) using HPLC. Statistical analysis was performed using two-way ANOVA with Tukey’s post-hoc test. * *p* < 0.05, ** *p* < 0.01, *** *p* < 0.001.

To determine changes that may occur in the intestinal environment, the fecal organic acid content due to intestinal flora activity (acetic acid, propionic acid, n-butyric acid, lactic acid, succinic acid, formic acid, n-valeric acid, and iso-valeric acid) was measured two days after IDM administration using high-performance liquid chromatography (HPLC) (Figure 5). The mean acetic acid content per 1 g of feces was 3.82 mg in the C group, 1.82 mg in the IDM group, and 3.5 mg in the IDM + H_2_RA group. The mean propionic acid content per 1 g of feces was 0.54 mg in group C, 0.42 mg in the IDM group, and 0.67 mg in the IDM + H_2_RA group. The mean n-butyric acid content per 1 g of feces was 1.07 mg in the C group, 0.13 mg in the IDM group, and 0.46 mg in the IDM + H_2_RA group. Therefore, the fecal levels of acetic acid, propionic acid, and n-butyric acid increased with IDM administration and decreased with the concurrent H_2_RA administration. Fecal lactic acid levels showed the same tendency as the organic acids mentioned above; however, there was no significant difference observed. Succinic acid, formic acid, n-valeric acid, and iso-valeric acid showed no change.

### 2.6. The Role of the Protective Factor in the Intestinal Mucosa

**Figure 6:**
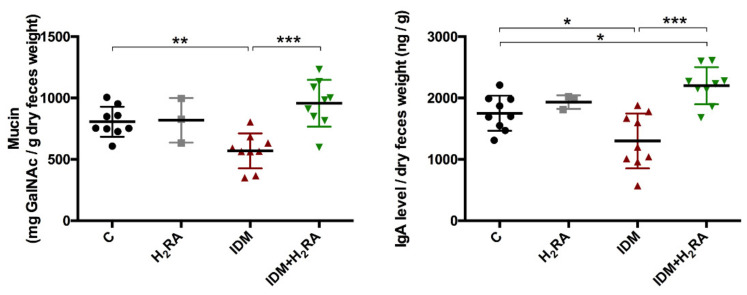
Changes in mucosal defense factors in the feces of Indometacin (IDM)-induced intestinal mucosal injury model with or without histamine H_2_-receptor antagonists (H_2_RA) administration. Two days after IDM administration, mucin and IgA levels were measured using the feces of 30 mice (9 control, 3 H_2_RA only, 9 IDM, and 9 IDM + H_2_RA mice). Statistical analysis was performed using two-way ANOVA with Tukey’s post-hoc test. * *p* < 0.05, ** *p* < 0.01, *** *p* < 0.001.

To examine the effects of IDM and H_2_RA on the intestinal barrier function, fecal mucin and secretory Immunoglobulin A (IgA) levels were measured (Figure 6). On the second day after IDM administration, the mean volume of mucin per 1 g of feces was 850 mg in group C, 570 mg in the IDM group, and 957 mg in the IDM + H_2_RA group. The mean volume of IgA per 1 g of feces was 1752 ng in group C, 1600 ng in the IDM group, and 2202 ng in the IDM + H_2_RA group.

Therefore, fecal mucin and IgA levels increased with IDM administration and decreased with the concurrent H_2_RA administration, which suggested that intestinal barrier function reduced by IDM administration was restored by the concurrent use of H_2_RA.

### 2.7. The Effect on the Intestinal Tissues

**Figure 7:**
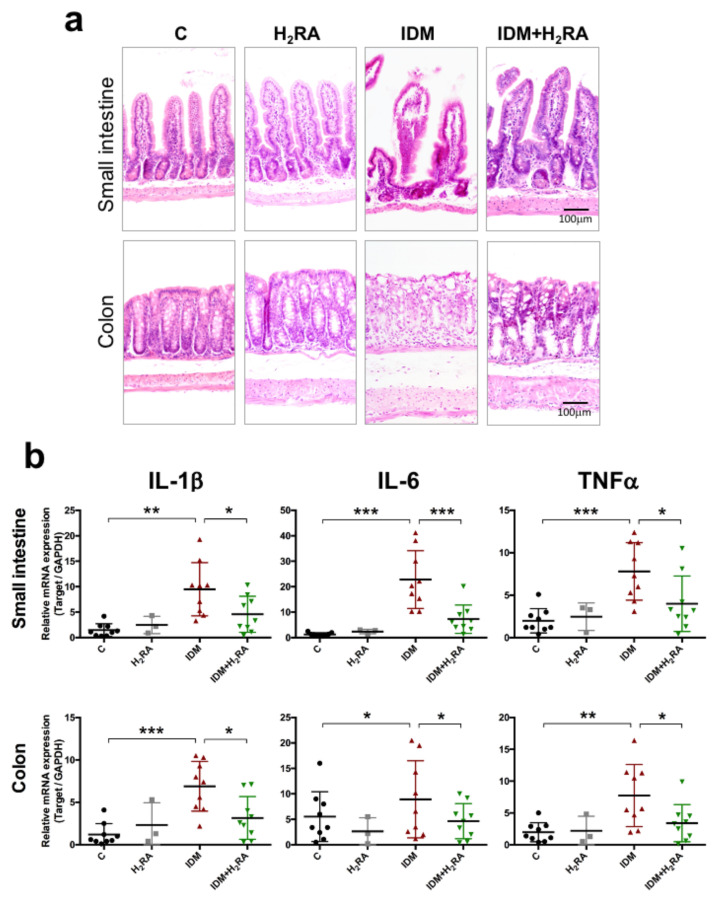
Morphological changes and effects of the immunologic reaction in the intestinal mucosa of the Indometacin (IDM)-induced intestinal mucosal injury model with or without histamine H_2_-receptor antagonists (H_2_RA) administration. Two days post-IDM administration, histological observation using H & E staining (**a**) and analysis of mRNA expressions of the IL-1β, IL-6, and TNFα (**b**) were performed using the intestinal tissue of 30 mice (9 control, 3 H_2_RA only, 9 IDM, and 9 IDM + H_2_RA mice). Statistical analysis was performed using a two-way ANOVA with Tukey’s post-hoc test. * *p* < 0.05, ** *p* < 0.01, *** *p* < 0.001.

The histologic heterogeneity of the jejunal villi and crypt, and cell infiltration increased in the IDM group compared with the C group (Figure 7a). On the other hand, in the IDM + H_2_RA group, the tissue damage was alleviated. No significant tissue damage was observed with H_2_RA alone. In the colon, similar changes were observed. Next, we focused on the dynamics of cytokines as inflammatory mediators and studied changes in the mRNA expression of cytokines. Gene expression in each tissue was analyzed by real-time polymerase chain reaction (PCR). Interleukin (IL)-6 mRNA expression was detected at low levels in the small intestine and colon of the C group. On the other hand, the mRNA expression was up-regulated by several to about 10 times in the IDM group. Similar to IL-6, relatively low levels of IL-1β and Tumor Necrosis Factor α (TNFα) mRNA expression were detected in the C group, but these expressions were up-regulated by several times in the IDM group (Figure 7b). On the other hand, in the IDM + H_2_RA group, gene expression was down-regulated. These results suggest that treatment with H_2_RA exerted an inhibitory effect on inflammation of the gastrointestinal mucosal tissue in IDM-induced intestinal disorders, increasing the defense function of epithelial cells.

## 3. Discussion

Studies have reported that dysbiosis of the intestinal flora is related to obesity and inflammatory bowel disease (IBD), as well as serious conditions such as colon cancer, liver cancer, and pneumonia. In patients, the composition of the intestinal flora changes and differs significantly from that of healthy individuals. Incidentally, the oral intake of non-steroidal anti-inflammatory drugs (NSAIDs) frequently causes injury to the gastrointestinal mucosa, and in the pathogenesis of NSAIDs/aspirin-induced small bowel injury, it has been found that the intestinal flora and, in particular, Gram-negative bacteria stimulate the innate immune system characterized by Toll-like receptor 4, thereby inducing inflammation [20]. The oral administration of Proton pump inhibitors (PPIs), which are widely used in clinical practice, causes dysbiosis of the intestinal flora, can exacerbate NSAID-induced small bowel injury [13]. Consequently, new therapeutic drug trials for candidates with probiotic and prebiotic mechanisms are anticipated, and evidence of such treatments is urgently being accumulated. Therefore, we focused on histamine H_2_-receptor antagonists (H_2_RA), which increases intestinal protection, and we examined the effect of H_2_RA on the intestinal flora for NSAID-induced intestinal mucosal injury.

First, on observing model mice prepared by NSAIDs administration during the rearing period, we found that Indometacin (IDM) administration had a physical impact such as weight loss, which improved with the concurrent administration of H_2_RA (Figure 1). As the underlying mechanism of this, it is conceivable that suppression of gastric acid secretion resulted in increased appetite, and increased the efficiency of nutrient absorption, which led to weight gain, or that H_2_RA directly affected intestinal cells and exhibited an anti-inflammatory action; however, in any case, we believe that mucosal injury caused by NSAIDs was histologically alleviated with the concurrent use of H_2_RA (not yet published) and led to recovery.

The fecal pH in the IDM group was slightly higher than weak acidity, which was returned to the weak acidity with the concurrent use of H_2_RA. Harmful bacteria, such as *Escherichia coli*, thrive in an alkaline environment but have difficulty surviving in an environment with a pH of <6 (weak acidity). This implies that IDM administration exacerbates conditions in the intestinal environment while H_2_RA improves them.

Bacteria belonging to the *Clostridiales* order are a genus of eubacteria and are Gram-positive obligate bacilli. Most bacteria detected in the human intestinal tract belong to the *Clostridiales* order. The *Clostridiales* order consists of many bacteria and inhabit abundantly in the intestinal tract. Furthermore, pathogenic bacteria that cause food poisoning such as botulinum bacillus, Welch bacillus, and tetanus bacillus are also included; therefore, bacteria of the *Clostridiales* order are often generally considered harmful bacteria [21,22,23]. However, it has been reported that some bacteria of the *Clostridiales* order that create butyric acid are effective against IBD [24]; therefore, in recent years, it has been thought that bacteria of the *Clostridiales* order are not necessarily harmful to the host, and can contrarily function in a favorable manner. In the present experiment, on inducing intestinal mucosal injury in the IDM group, the amount of bacteria of the *Clostridiales* order decreased and was restored in the IDM + H_2_RA group (Figure 3). This finding can be interpreted to indicate that mucositis (Figure 6) causes a reduction in butyric acid-producing bacteria of the *Clostridiales* order as good bacteria, and that immunity is activated by increasing butyric acid-producing bacteria of the *Clostridiales* order with the concurrent H_2_RA administration.

Bacteria of the *Erysipelotrichaceae* family are anaerobic Gram-positive bacilli, infecting pigs, wild boar, and birds presenting primary symptoms such as arthritis [25,26]. Furthermore, there are also cases of such bacilli isolated from human empyema and brain that have contracted infection, and on rare occasions, such bacilli are isolated from the blood and have affected parts of immunocompromized individuals [27]. However, there are few reports of such bacteria, and little is known about the bacteria. For example, from the little information known, *Clostridium innocuum*, which is classified into the *Erysipelotrichaceae* family (previously, it was classified into the *Clostridiales* order, but it presently belongs to the *Erysipelotrichaceae* family) is a vancomycin-resistant pathogen [28], with a reported case of endocarditis caused by this bacteria [29]. Thus, there is the view that it is a fatal bacterium that cannot be taken lightly. However, in an experiment with this bacterium, using rodents such as mice, infection and toxicity were not detected, and, therefore, future research and development of *Clostridium innocuum* as an unknown bacterium are anticipated, to determine the relationship with healthy humans and the role it plays in the intestines. Our data (Figure 3) indicates that as a result of damaging the intestinal mucosa and causing infection, bacteria of the family *Erysipelotrichaceae* proliferated, whereas the concurrent administration of H_2_RA inhibited infection; therefore, decreasing the amount of *Erysipelotrichaceae* family bacteria. Although it is presently unclear whether bacteria of the *Clostridiales* order, which act on cells in the immune system, and bacteria of the *Erysipelotrichaceae* family, which proliferate from reduced immunity, are mutually related, with regard to the dynamics of both, we believe that the results of the present study are very interesting.

Bacteria of the large intestine break down digestion-resistant saccharides, thereby producing short-chain fatty acids (SCFA) such as acetic acid and butyric acid, as well as organic acids such as lactic acid and succinic acid. Accordingly, to identify changes in the intestinal environment that inhibit intestinal bacteria, it is important to detect organic acids in feces. On inducing intestinal mucosal injury by IDM administration, acetic acid, propionic acid, butyric acid, and lactic acid levels decrease, which then were subsequently increased with the concurrent H_2_RA administration (Figure 5). To date, it has been reported that on feeding butyric acid to mice with colitis, colitis was inhibited, and therefore, butyric acid-producing *Clostridiales* induce T regulatory (Treg) cell differentiation [24], that propionic acid acts on thymus-derived Treg cells (tTreg), thereby exhibiting a colitis-inhibitory effect [30], that acetic acid promotes neutrophil apoptosis, thereby inhibiting colitis [31], and that acetic acid improves the barrier function of immune cells, as well as the single layer of intestinal epithelial cells covering the intestinal lumen [32], which demonstrates that SCFA and organic acids produced by metabolism and fermentation of the intestinal flora contribute to homeostasis of the host. In the present study, we believe that the concurrent administration of H_2_RA leads to increased SCFA and organic acid secretion and alleviates NSAID-induced mucosal injury. The underlying mechanism of this is a topic for future research. While there was no significant difference observed, the administration of H_2_RA alone reduced acetic acid, propionic acid, and lactic acid levels. This is thought to be attributed to the fact that H_2_RA has an antacid action, and therefore suppressing gastric acid indirectly affected the intestinal environment downstream. In fact, there was little difference in the composition of the intestinal flora between the control group and the H_2_RA monotherapy group (Figure 3), and this phenomenon can be interpreted to indicate no change caused by the intestinal flora. Furthermore, fecal succinic acid levels increased with IDM, and decreased with the concurrent H_2_RA, although there was no significant difference observed. Succinic acid is a metabolite produced in high concentrations by so-called bad bacteria such as bacteria of the *Bacteroidaceae* family. Moreover, it has been reported that succinic acid levels increased with the onset of obesity and colitis, which therefore suggests that it might be a metabolite that has a harmful effect on the host [33,34], and which also suggests that succinic acid levels produced by bad bacteria increased in number by NSAID-induced colitis that was inhibited by the concurrent H_2_RA administration.

To adjust the intestinal environment and maintain health, the system of removing pathogens and exogenous antigens must function. In the present experiment, the fecal levels of mucin and IgA decreased on inducing intestinal mucosal injury in the IDM group and were restored in the IDM + H_2_RA group (Figure 6). Our group has reported that lafutidine, which is one H_2_RA, promotes mucus secretion in the intestinal tract with mucositis [17,18], and in NSAID-induced mucosal injury, we examined mucin secretion as an indicator of the mucosal protective effect of H_2_RA. A difference compared to the previous report and methodology lies in the fact that we used feces as test samples. As expected, fecal mucin levels that were decreased by IDM administration increased on administration of H_2_RA, and the same phenomenon was found for fecal IgA levels, which prevent the invasion of microorganisms into the mucosal surface. Indeed, in this model, IDM administration exacerbated inflammation of mucosal tissue with an increased inflammatory cytokine expression, while combined use with H_2_RA alleviated these symptoms (Figure 7). This suggests that H_2_RA might help to strengthen the mucosal barrier at the time of injury.

As a prerequisite for using multiple drugs, it must be in a state where the effects of the drugs are fully realized. We have used different routes of administration for the two agents to avoid them from metabolically interfering with each other. So, we believe that the H_2_RA (Lafutidine) administration has almost no effect on the pharmacokinetics of IDM. In fact, cell viability in drug stimulation experiments using Caco-2 showed that there was almost no loss of cytotoxicity caused by IDM even with the addition of H_2_RA (Supplementary Figure S1). These results strengthen the assertion that H_2_RA does not offset the pharmacological effects of IDM.

Certainly, H_2_RA administered to individuals without underlying enteritis, such as those of very young age, can contrarily cause inflammation as indicated by reports that H_2_RA administration to infants with low-birth-weight causes enteritis [35] and that administration of antacids in infancy causes obesity [36]. Thus, caution is advised with the use of H_2_RA in treating infants and children. Therefore, H_2_RA, like PPI, can exacerbate inflammation; however, compared to aspirin combined with H_2_RA, when PPIs are combined with aspirin, the rates of recurrent hemorrhage and ulcer onset increase [37], which therefore suggests that the intestinal environment-improving effect of H_2_RA might help to treat enteritis. However, the mechanism underlying the inflammation-alleviating effect remains a topic for future research. H_2_RA should be used cautiously in patients with IBD [38,39,40]. It is suggested that intestinal flora contributes to the pathology of IBD, in that patients with IBD show a decrease in the diversity of the intestinal flora, and that the pathology involves the breakdown of intestinal homeostasis caused by bacteria of the Clostridium family. The bacterial composition of dysbiosis in the present NSAID-induced gastrointestinal injury and IBD differs from the composition of intestinal flora (Figure 3). We believe that the intestinal environment differs depending on the underlying mucosal damage.

Furthermore, in the present experiment, we did not use living constituents such as tissue or cells, and the results were obtained using indirect (non-invasive) samples, i.e., feces. Therefore, we were able to evaluate the effect of H_2_RA in a more objective manner and using minimally invasive samples will help for clinical introduction.

## 4. Materials and Methods

### 4.1. Overall Experimental Design and Evaluation Methods

The mucosal injury murine model was prepared using NSAIDs. First, the degree of injury and the systemic effects of the drug combination were evaluated by biological observation. In this study, we used noninvasive samples to evaluate the likelihood of clinical factors, and only feces were used as experimental samples. We evaluated the effect of the drug using fecal properties, analyses of intestinal bacterial flora and organic acid, and measurements of various proteins and mucin. All of the experiments were performed according to the Institutional Guidelines for the Care and Use of Laboratory Animals in Research and were approved by the local ethics committee of Kitasato University (approval number: 17-06-3, approval date: 26 April 2019, approver: Hidero Kitasato, Ph.D.; Chairperson of School of Allied Health Sciences, Kitasato University).

### 4.2. Mice and Drug Treatment

We obtained 7-week-old male BALB/cAJcl mice (CLEA-Japan, Tokyo, Japan). The number of mice allocated to each group was 2–16, depending on the experimental systems shown in each figure legend. All of the mice were bred under specific pathogen-free conditions at the School of Allied Health Sciences, Kitasato University, Japan. All of the animals were maintained at 23 ± 3 °C in a 12-h light–dark cycle. The mice were provided a commercial diet (CRF-1, Oriental Yeast Co., Ltd., Tokyo, Japan) and water ad libitum. All of the experiments were performed according to the Institutional Guidelines for the Care and Use of Laboratory Animals in Research and were approved by the local ethics committee at Kitasato University. Indomethacin (IDM) (Merck & Co., Inc., Darmstadt, Germany) as a NSAID was administered by subcutaneous injection once (30 mg/kg). IDM was dissolved in 0.03 M NaOH, neutralized with 0.05 M HCl, and suspended in saline. Control animals received saline instead of IDM. Lafutidine (Taiho Pharmaceutical Co., Ltd., Tokyo, Japan) as H_2_RA was administrated orally by gavage (30 mg/kg/day) once daily for 2 days. H_2_RA was suspended in 0.5% carboxymethylcellulose (CMC) solution and was prepared immediately before use. The control animals received 0.5% CMC instead of H_2_RA. IDM was administered once on the first day (Day 0), and H_2_RA was administered twice on the first day (Day 0) and the next day (Day 1). H_2_RA administration on day 0 occurred 6 h after IDM administration, and H_2_RA administration on day 1 occurred 24 h after the first H_2_RA administration. Body weight, stool volume, and food consumption were measured before the administration of H_2_RA on days 0–2 once a day. All of the experimental procedures, such as oral administration and stool collection, were conducted by researchers skilled in handling mice. In addition, the biological observation as well as experimental and data analyses were performed by researchers with 10 or more years of training.

### 4.3. Observation of Feces

The collected feces were observed visually, and the length of the feces was measured. The weight of each stool sample was measured. The feces were freeze-dried and weighed again to calculate the water content percentage. The freeze-dried feces were crushed in distilled water, and the pH was measured using a pH meter (Horiba, Ltd., Kyoto, Japan).

### 4.4. Tests of the Intestinal Microbiome

The feces (40–50 mg/mouse; wet weight) collected on the second day after the administration of drugs were placed into tubes and stored at −80 °C until analysis. The collected feces were homogenized, and a portion of the homogenate was suspended using a stool collection kit (TechnoSuruga Laboratory Co., Ltd., Shizuoka, Japan); then, DNA was extracted using the bead-phenol method. For the 16S rRNA derived from the bacteria, we performed terminal restriction fragment length polymorphism (T-RFLP) analysis using the extracted DNA as a sample, and the estimates and proportions of the bacterial flora-forming group existing in the feces were determined and compared between each group. ABI PRISM 310 Genetic Analyzer (Applied Biosystems, Waltham, MA, USA) and GeneScan (Applied Biosystems, Waltham, MA, USA) were used for the analysis. The lengths of each fragment were determined by OTU (operational taxonomic unit), and the major taxonomic groups were estimated on the basis of the murine intestinal bacterial flora database. The detection and analysis by T-RFLP used the commissioned enteral bacterial flora inspection service of the CIEA “ICLAS Monitoring Center” (Central Institute for Experimental Animals, Kanagawa, Japan).

### 4.5. Fecal Mucin Assay

The amounts of mucins in the feces were measured using Fecal Mucin Assay Kit (Cosmo Bio Co., Ltd., Tokyo, Japan). The amount of mucin in the feces was obtained by decomposing the o-glycan in mucin structure by β-elimination under alkaline conditions, simultaneously and fluorescently labeling the reducing end of the sugar chain and measuring its fluorescence intensity. Their fluorescence intensities (Em 383 nm and Ex 336 nm) were measured using a microplate reader (Molecular Devices, San Jose, CA, USA).

### 4.6. Fecal Organic Acid Analysis

The feces (50–70 mg/mouse; wet weight) collected on the second day after the administration of the drugs were immediately placed in tubes and stored at −80 °C until needed. The samples were heat-treated at 85 °C for 15 min. The samples were then crushed with beads, centrifuged at 14,000 rpm for 10 min, and the supernatants were filtered through a membrane filter with a pore size of 0.20 μm to yield the sample solutions. The concentration of each organic acid contained in the sample was measured using HPLC after pretreatment. The organic acids, which were analyzed, comprised nine substances: succinic, lactic, formic, acetic, propionic, iso-butyric, n-butyric, iso-valeric, and n-valeric acids. The detection and analysis of fecal organic acid used the commissioned enteral bacterial flora inspection service of the CIEA “ICLAS Monitoring Center” (Central Institute for Experimental Animals, Kanagawa, Japan).

### 4.7. Enzyme-Linked Immunosorbent Assay (ELISA)

The freeze-dried feces samples were crushed and dissolved in 100 times the amount of phosphate-buffered saline containing protease inhibitors (Thermo Scientific, Waltham, IL, USA). Immunoglobulin A (IgA) in the feces was measured using an ELISA kit (Bethyl Laboratories, Inc., Montgomery, TX, USA), according to the manufacturer’s instructions.

### 4.8. Histologic Analysis

The tissues were immediately fixed for 24 h in freshly prepared 4% paraformaldehyde in PBS. After fixation, 4 µm paraffin sections were stained with hematoxylin-eosin (H&E). An optical microscope was used to observe and photograph the tissues. Evaluation of the tissue image was performed using an optical microscope (Olympus Corporation, Tokyo, Japan).

### 4.9. Real-Time Reverse Transcription-Polymerase Chain Reaction

Transcripts encoding IL-1β, IL-6, TNFα, and Glyceraldehyde-3-phosphate dehydrogenase (GAPDH) were determined by real-time reverse-transcription polymerase chain reaction (RT–PCR). Briefly, total RNA was purified from the tissues using the TRIzol RNA Isolation Reagents (Thermo Fisher Scientific, Waltham, MA, USA). Single-stranded cDNA was generated from the total RNA by reverse transcription using the PrimeScript RT reagent Kit (TAKARA BIO INC., Shiga, Japan), according to the manufacturer’s instructions. Quantitative PCR amplification was performed with SYBR Select Master Mix (Thermo Fisher Scientific, Waltham, MA, USA). The gene-specific primers used were as follows: IL-1β, 5′-gggctgcttccaaacctttg and 5′-aagacacaggtagctgccac; IL-6, 5′-agttgccttcttgggactga and 5′-tccacgatttcccagagaac; TNFα, 5′-gacgtggaactggcagaaga and 5′-actgatgagagggaggccat; GAPDH, 5′-tgatgggtgtgaaccacgag and 5′-agtgatggcatggactgtgg. Data were normalized to the level of GAPDH in each sample.

### 4.10. Cell Culture and Cell Viability Assay

Cell cultures of Caco-2 cells, obtained from a human colonic carcinoma, were grown in a culture medium comprising of DMEM with 4 mmol/L glutamine, and 10% FBS in a 10 cm^2^ tissue culture dish. The cells were maintained at 37 °C in an atmosphere of 5% CO_2_/95% air in a CO_2_ incubator. The cells were sub-cultured by partial digestion with 0.25% trypsin and 1 mmol/l EDTA in Ca^2−^ and Mg^2−^ free phosphate-buffered saline (PBS) solution.

The Caco-2 cells (passage 5–7) were plated on a 96 well culture plate (1 × 10^5^ cells/well). After 24 h, IDM and/or H_2_RA were added to the culture supernatant (IDM, 0, 10, 100, or 1000 ng/mL final conc. and H_2_RA, 0, 10, 50, or 100 μm/mL final conc.) and cultured for 12, 24, and 48 h. The viability of Caco-2 cells was determined by a cell proliferation assay using MTT reagent (Nacalai Tesque, Inc., Tokyo, Japan). An MTT solution was added to each well. Following a 3 h incubation at 37°C, a microplate reader (Bio-Rad Laboratories, Hercules, CA, USA) was used to determine the absorbance at 570 nm (reference at 650 nm). Percentage cell viability was calculated based on the absorbance measured relative to that of cells not exposed to the MTT solution.

### 4.11. Statistics

The data were expressed as mean ± SD values. A statistical analysis was performed using GraphPad Prism version 6.0 (GraphpPad Software, Inc., San Diego, CA, USA). Multiple comparison tests were performed using Tukey’s post-hoc test. The normality of the distribution of continuous variables was evaluated using the Kolmogorov–Smirnov test. Most data were confirmed using the post-hoc sample size power calculation to verify whether there was adequate power to evaluate the outcomes studies. However, in the case of an experiment with a small number of samples, they were distinguished and indicated each time.

## 5. Conclusions

We found that in gastroenteritis caused by Non-Steroidal Anti-Inflammatory Drugs (NSAIDs), the proportion of enteric microbe’s changes, which might lead to deterioration of the intestinal environment, and that the concurrent use of histamine H_2_ receptor antagonist (H_2_RA) can normalize the intestinal flora.

## Supplementary Materials

Supplementary Materials can be found at https://www.mdpi.com/1422-0067/21/21/8166/s1, Figure S1: Pharmacological interference experiments of IDM and H_2_RA on intestinal epithelial cells.

## Abbreviations

H_2_RA	histamine H_2_ receptor antagonist
NSAIDs	Non-Steroidal Anti-Inflammatory Drugs
IDM	Indometacin
T-RFLP	Terminal Restriction Fragment Length Polymorphism
OTU	Operational Taxonomic Unit
Ig	Immunoglobulin
IL	Interleukin
TNF	Tumor Necrosis Factor
