# Supplementary material for: Histamine H2-Receptor Antagonists Improve Non-Steroidal Anti-Inflammatory Drug-Induced Intestinal Dysbiosis
Source: Int J Mol Sci. 2020 Oct 31;21(21):8166. doi: 10.3390/ijms21218166 (PMC7662336; doi:10.3390/ijms21218166)
Supplement: Supplementary file 1 [file ijms-21-08166-s001.pdf]

Supplementary Figure

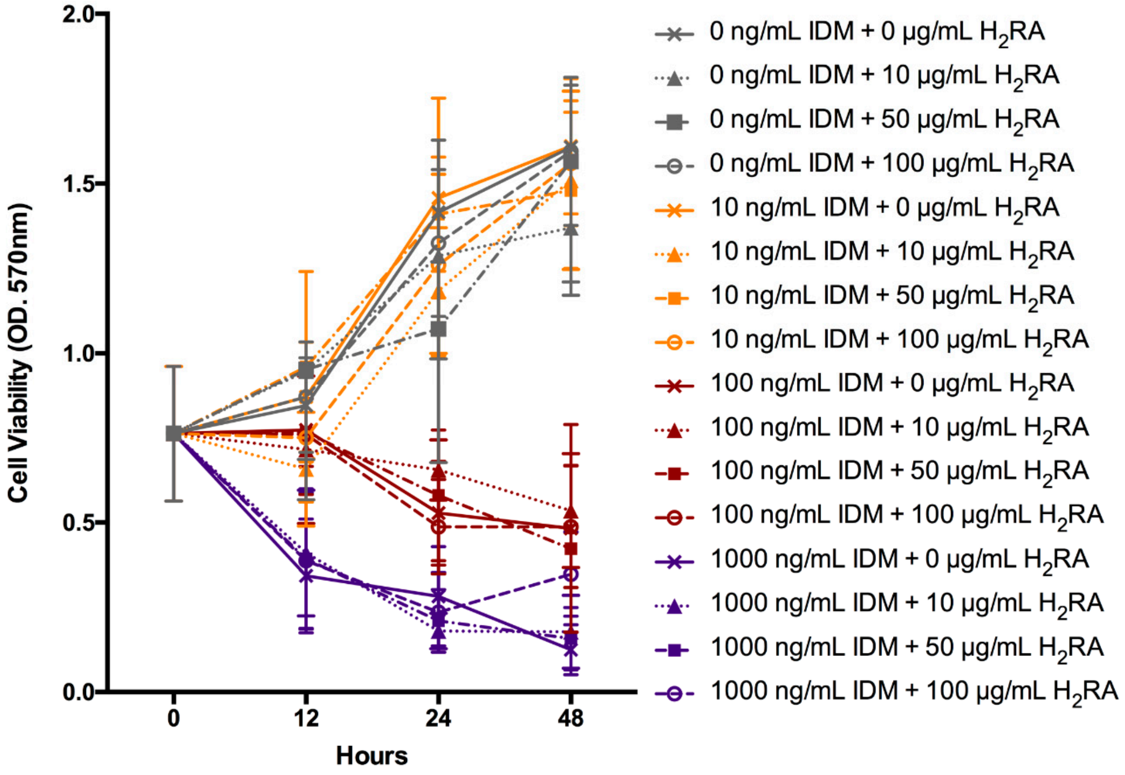

Supplementary Figure S1. Pharmacological interference experiments of Indometacin (IDM) and histamine H_2-receptor antagonists (H_2RA) on intestinal epithelial cells. The cell viability of the human colonic carcinoma cell line Caco-2 subjected to IDM and H_2RA was evaluated. Statistical analysis was performed using a two-way ANOVA with Tukey’s post-hoc test. *$p < 0.05$, **$p < 0.01$, ***$p < 0.001$.
